# *H. pylori* Infection and Colorectal Cancers by Anatomical Locations

**DOI:** 10.31557/APJCP.2020.21.8.2431

**Published:** 2020-08

**Authors:** Dao Viet Hang, Dinh Thi Minh, Tran Hieu Hoc, Le Hong Phuoc, Tran Que Son, Ngoan Tran Le

**Affiliations:** 1 *Department of Internal Medicine, Hanoi Medical University, Hanoi, Vietnam. *; 2 *Institute of Gastroenterology and Hepatology, Hanoi, Vietnam. *; 3 *Department of Surgery, Hanoi Medical University, Hanoi, Vietnam. *; 4 *Faculty of Public Health, University of Medicine and Pharmacy at Ho Chi Minh City, Vietnam. *; 5 *Institute of Research and Development, Duy Tan University, Da Nang 550000, Vietnam. *; 6 *Department of Public Health, School of Medicine, International University of Health and Welfare, Japan. *

**Keywords:** Colorectal cancer, H. pylori, case-control study

## Abstract

**Background:**

*H. pylori* infection may play a role in the development of colorectal cancers (CRC). We aimed to examine the association between *H. pylori* infection and the risk of CRC by anatomical locations.

**Methods:**

We conducted a case-control study on 91 incidence cases of CRC and 224 hospital controls. CRC was determined by histopathological examinations. *H. pylori* IgG antibody in serum was tested. We collected data on the diet, nutrition, and lifestyle by the validated semi-quantitative food frequency and demographic lifestyle questionnaire. The odds ratio and 95% confidence interval (OR (95%CI) were estimated for CRC and its subgroups.

**Results:**

Overall 54.95% of CRC cases and 42.41% of the controls were *H. pylori*-seropositive, OR (95%CI): 1.56 (0.88, 2.74), p for trend=0.115. Positive dose-response association in quartiles, highest vs lowest, was observed for total CRC, OR (95%CI): 2.14 (1.00, 4.58), p for trend=0.049, for proximal colon, OR (95%CI): 1.52 (0.37, 6.25), p for trend=0.571), and for distal colon and rectum cancers combined, OR (95%CI): 2.38 (1.03, 5.50), p for trend=0.039.

**Conclusions:**

There is a positive association between *H. pylori* and colorectal cancers, especially distal colon and rectum cancers combined, but additional research is needed to determine the underlying mechanism of chronic *H. pylori* infection-induced CRC in humans.

## Introduction

For many decades, the standard triple therapy to eradicate *H. pylori* infection and prevent gastric cancer, including a proton pump inhibitor combined with clarithromycin and amoxicillin or metronidazole, has been recommended in the Vietnamese and other populations for both children and adults (Nguyen et al., 2008; Papastergiou et al., 2014). However, the antibiotic regimen in *H. pylori* treatment is likely to affect the digestive microbiome (Langdon et al., 2016; Modi et al., 2014), as well as resistance rate, has been increasing in recent years (Khien et al., 2019). Widespread antibiotic use has significant implications for public health and their effects on gut microbiota. Antibiotic use is common and gradually increasing in Vietnam where people can buy easily in market pharmacy stores. That is, there were about an estimated 10 defined daily doses of antibiotic use per person per year in Vietnam and worldwide in 2015 (CDDEP, 2019). Antibiotic use predicts an increased risk of cancers (Kilkkinen et al., 2008). Oral use of antibiotics associated with the increased risk of colon cancer in the U.K (Zhang et al., 2019). Also, the use of antibiotics during 24-59-year-old was associated with the increased risk of colorectal adenomas (CRA), precursors of colorectal cancers (CRC) (Cao et al., 2017). There are differences in the gut microbiome between right and left colon. Bacteria gram-negative of *Fusobacterium, Escherichia/Shigella*, and *Leptotrichia* are more abundant in the left than right colon (Kim et al., 2018). *H. pylori* is also a gram-negative bacteria, therefore, the standard triple therapy to eradicate *H. pylori*, including a proton pump inhibitor combined with clarithromycin and amoxicillin or metronidazole, might affect to these groups of gram-negative bacteria of *Fusobacterium, Escherichia/Shigella*, and* Leptotrichia *colonized in the distal colon. We have hypothesized that *H. pylori* infection might highly target distal colon cancer. 

Antibiotic use significantly alters the compositions of gut microbiota and might impair the integrity of the intestinal barrier (Tulstrup et al., 2015); thus, it allows for colonization of carcinogenic bacteria, such as *H. pylori*, that induces local inflammation and tumor formation in CRC. In a study on 100 patients who had undergone a colonoscopy, 22% had DNA evidence of *H. pylori* colonization (Keenan et al., 2010). In another case-control study, 40% of the controls and 41% of the CRC cases were *H. pylori*-seropositive and serologic response to *H. pylori* proteins was associated with CRC among Africa Americans but not in another ethnic group (Butt et al., 2019). Results of nine Meta-analyses (Chen et al., 2013; Guo & Li, 2014; Hong et al., 2012; Rokkas et al., 2013; Wu et al., 2013; Yang et al., 2019; Zhao et al., 2016; Zhao et al., 2008; Zumkeller et al., 2006) for the published studies during 1991-2018, showed that there was a positive association between *H. pylori* infection and CRC.

Although results from the previous studies on the association between *H. pylori* infection and CRC are inconsistent. This inconsistency could be attributable to the quality of the previous observational studies when the data related to the CRC of diet and nutrition and smoking status (IARC, 1986) was not included as confounding factor adjustment in the data analysis due to their limitation of the available database (Butt et al., 2019; Epplein et al., 2013; Liu et al., 2019; Park et al., 2018; Strofilas et al., 2012; Teimoorian et al., 2018). 

Colorectal cancers are an important public health issue throughout the world, especially in Asian countries including Vietnam. The latest data on Globocan 2018 reported that in both sexes, colorectal cancers was the third leading incidence worldwide with the estimated number of 1,849,518 (10.2% of 18,078,957 cases). Asian countries contributed to nearly half of the estimated number of CRC (8,750,932 new cases, giving 48.4% of the total CRC in the world). In 2018, Vietnam had the estimated number of 14,733 new CRC cases (8.9% of 164,671) and CRC ranked the fifth most common cancer (IARC, 2019). Vietnam is located in Southeast Asia and had a population of over 96 million in 2019 (The National Census, data in press). The estimated prevalence rate of *H. pylori* infection in Vietnam was common, as high as about 60%-80% of the general population or specific study populations (Binh et al., 2017; Binh et al., 2018; Hoang et al., 2006; Megraud et al., 1989; Nguyen et al., 2006; Nguyen et al., 2010; Nguyen et al., 2012; Truong et al., 2009). Therefore, *H. pylori* infection may play a role in the development of CRC, especially distal colon and rectum cancers in Vietnam.

## Materials and Methods

*Study population*

**Figure 1:**
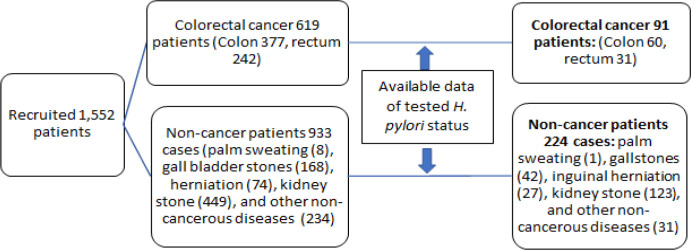
Flow-Chart of the Recruitment of Colorectal Cancer Cases and Non-Cancer Controls

We conducted a case-control study on 91 colorectal cancers (CRC) and 224 hospital controls treated at Bach Mai hospital, Hanoi, during 2017-2018. The incidence case of CRC was determined by histopathological examinations. We periodically reviewed the weekly list of patients admitted to the department of General Surgery of Bach Mai hospital for a planned operation and selected cases of CRC and then selected controls, matching for age (+/- 5-year-old) and sex. From December 2017 to December 2018, among 619 CRC patients admitted to the study site, 91 cases had data on *H. pylori* infection. Among 933 matched controls recruited, 224 cases had data on *H. pylori* infection. Surgery indications included palm sweating (1), gallstones (42), inguinal herniation (27), kidney stone (123), and other non-cancerous diseases (31). Finally, 91 CRC and 224 control patients participated in the present study (Figure 1).

*Examination of H. pylori infection*

To test for antibodies to *H. pylori*, 3 ml aliquots of overnight fasting blood was collected from cases and controls who had undergone surgery in the Department of General Surgery of Bach Mai hospital. Among 1,552 patients, we only collected blood samples and tested successfully for 91 CRC patients and 224 controls due to intensive care during they were in the in-patient department. *H. pylori* IgG antibody in serum was examined. The anti-*H. pylori* serum IgG titers were tested by the enzyme-linked immunosorbent assay (ELISA) using *H. pylori* IgG ELISA kit (RE56381) (IBL International, Hamburg, Germany). All plasma samples were tested by the experienced microbiologists. According to the manufacturer’s instructions, *H. pylori* serostatus was classified into three groups based on the Cut-Off Index (COI) including negative (COI <0.8), positive (COI >1.2) and equivocal (0.8-1.2). We also categorized the qualitative results of anti-*H. pylori* IgG concentration into quartiles to examine for a dose-response for the association between *H. pylori* infection and CRC and its subgroups. 

*Covariation of possible confounding factors*

Two confounding factors often included in analysis models in studies on CRC were smoking and nutrition status (Cao et al., 2017; Le et al., 2016). To collect information on these factors, we used the validated questionnaires of semi-qualitative food frequency questionnaires and demographic lifestyle questionnaire. For tobacco smoking, we classified participants into two categories: never-smokers and ever-smokers (including former smokers and current smokers). Never-smokers were those who never smoked completely only one cigarette or waterpipe tobacco (WPT) smoking in their lifetime. For smokers, the information on types of tobacco (cigarette, WPT, or both types), the average number of cigarette and WPT per day during their age of 15-20, 21-25, 26-30, 31-40, 41-50, 51-60, 61-70, and 71+ (if applicable), the duration of smoking (current smokers), and the duration of quit smoking (current and former smokers) were obtained. 

We collected data on diet, nutrition, and lifestyle by the validated semi-quantitative food frequency and demographic lifestyle questionnaire (SQFFQ). Dietary history in the last 12 months from the date of the interview was collected by trained interviewers. The designed SQFFQ comprised of 85 foods/items of 12 groups of foods/recipes. We classified the food frequencies of intake into seven categories: never or seldom, 6-11 times/year, 1-3 times/month, 1-2 times/week, 3-4 times/week, 5-6 times/week, and 1-3 times/day (Le et al., 2018).

*Data collection and management*

We prepared the designed questionnaires into electronic databases in using a smart tablet for data entry. The trained interviewers collected data via direct interviews at the in-patient department using the designed SQFFQ. The obtained data then uploaded to the designed website for management, editing variables, cleaning, and merging with the data on *H. pylori* infection status and the result of histopathological examinations for both cancer and control cases.

*Data processing and statistical analysis*

Collected data was exported into STATA 10 for cleaning and analysis. The crude and adjusted odds ratio and 95% confidence interval (OR (95%CI) were estimated controlling for possible confounding factors for CRC and the subgroups by unconditional logistic regression analysis. Target associations were adjusted for age groups (0-29, 30-39, 40-49, 50-59, 60-69, and ≥70 years), sex, BMI (<18.5, 18.5 to <23, 23 to <25, and ≥25 kg/m^2^), an education level (primary school or under, secondary school, high school, above high school, and unknown), and lifetime smoking (yes/no), total fruits intake (tertile), total vegetables intake (tertile), total meats intake (tertile), total fishes intake (tertile). All p-values were two-sided, and p ≤ 0.05 (alpha value) was considered to indicate statistical significance.

*Ethical approval*

The study was approved by the institutional review board at Hanoi Medical University (certificate of approval issued on December 25, 2018). We obtained written informed consent from all participants in the study.

**Table 1:** *H. pylori* Infection Characteristics

Variables	Total n (%)	Mean ± SD ^a^	Min-max ^a^
*H. pylori* infection status ^b^			
Negative (<0.8)	118 (37.46)	0.50 ± 0.16	0.13 - 0.79
Equivocal (0.8-1.2)	52 (16.51)	1.00 ± 0.11	0.81 - 1.20
Positive (>1.2)	145 (46.03)	2.35 ± 0.93	1.21 - 5.33
Anti-*H. pylori *IgG concentration quartiles ^c^			
1^st^	81 (25.71)	0.42 ± 0.11	0.13 - 0.59
2^nd ^	85 (26.98)	0.85 ± 0.17	0.60 - 1.14
3^rd ^	76 (24.13)	1.59 ± 0.27	1.15 - 2.04
4^th ^	73 (23.17)	3.08 ± 0.76	2.07 - 5.33

^a^, Mean ± SD, Mean ± Standard deviation; Min-Max, Minimum - Maximum; ^b^, Classification according to the manufacturer's instructions; ^c^, Classification according to anti-H. pylori IgG concentration of the Cut-off Index (COI) qualitative

**Table 2:** Crude and Adjusted OR and 95% CIs of *H. pylori* Infection and the Risk of Colon Cancer

Variables	Case		Control		Crude OR^a^	p for trend	Adjusted OR	p for trend ^b^
	n	%	n	%	(95% CI)		(95% CI)^b^	
Colon cancer								
*H. pylori *infection status^ c^								
Negative (<0.8)	18	30	90	40.18	1.00 (reference)	0.087	1.00 (reference)	0.212
Equivocal (0.8-1.2)	9	15	39	17.41	1.15 (0.48, 2.79)		1.00 (0.39, 2.53)	
Positive (>1.2)	33	55	95	42.41	1.74 (0.91, 3.30)		1.52 (0.77, 3.01)	
Total	60	100	224	100				
Anti-*H. pylori* IgG concentration quartiles ^d^								
1^st ^	11	18.33	66	29.46	1.00 (reference)	0.04	1.00 (reference)	0.142
2^nd ^	15	25	60	26.79	1.50 (0.64, 3.52)		1.32 (0.54, 3.24)	
3^rd ^	16	26.67	52	23.21	1.85 (0.79, 4.32)		1.75 (0.71, 4.32)	
4^th ^	18	30	46	20.54	2.35 (1.01, 5.43)		1.84 (0.76, 4.47)	
Total	60	100	224	100				
Proximal colon cancer								
*H. pylori* infection status ^c^								
Negative (<0.8)	5	22.73	90	40.18	1.00 (reference)	0.312	1.00 (reference)	0.409
Equivocal (0.8-1.2)	7	31.82	39	17.41	3.23 (0.97, 10.81)		2.59 (0.72, 9.28)	
Positive (>1.2)	10	45.45	95	42.41	1.89 (0.62, 5.76)		1.74 (0.53, 5.70)	
Total	22	100	224	100				
Anti-*H. pylori* IgG concentration quartiles ^d^								
1^st ^	4	18.18	66	29.46	1.00 (reference)	0.329	1.00 (reference)	0.571
2^nd ^	7	31.82	60	26.79	1.93 (0.54, 6.90)		1.56 (0.40, 6.03)	
3^rd ^	5	22.73	52	23.21	1.59 (0.41, 6.21)		1.87 (0.42, 8.28)	
4^th ^	6	27.27	46	20.54	2.15 (0.57, 8.06)		1.52 (0.37, 6.25)	
Total	22	100	224	100				
Distal colon and rectal cancers combined								
*H. pylori* infection status ^c^								
Negative (<0.8)	23	33.33	90	40.18	1.00 (reference)	0.077	1.00 (reference)	0.134
Equivocal (0.8-1.2)	6	8.7	39	17.41	0.60 (0.23, 1.59)		0.58 (0.22, 1.57)	
Positive (>1.2)	40	57.97	95	42.41	1.65 (0.91, 2.97)		1.54 (0.84, 2.81)	
Total	69	100	224	100				
Anti-*H. pylori* IgG concentration quartiles ^d^								
1^st ^	11	15.94	66	29.46	1.00 (reference)	0.015	1.00 (reference)	0.039
2^nd ^	18	26.09	60	26.79	1.80 (0.79, 4.12)		1.72 (0.74, 3.98)	
3^rd ^	19	27.54	52	23.21	2.19 (0.96, 5.01)		2.15 (0.92, 4.99)	
4^th ^	21	30.43	46	20.54	2.74 (1.21, 6.22)		2.38 (1.03, 5.50)	
Total	69	100	224	100				
Rectal cancer								
*H. pylori* infection status ^c^								
Negative (<0.8)	10	32.26	90	40.18	1.00 (reference)	0.246	1.00 (reference)	0.267
Equivocal (0.8-1.2)	4	12.9	39	17.41	0.92 (0.27, 3.12)		0.99 (0.29, 3.40)	
Positive (>1.2)	17	54.84	95	42.41	1.61 (0.70, 3.70)		1.60 (0.68, 3.76)	
Total	31	100	224	100				
Anti-*H. pylori* IgG concentration quartiles ^d^								
1^st ^	4	12.9	66	29.46	1.00 (reference)	0.091	1.00 (reference)	0.129
2^nd ^	10	32.26	60	26.79	2.75 (0.82, 9.23)		2.66 (0.78, 9.06)	
3^rd ^	8	25.81	52	23.21	2.54 (0.72, 8.90)		2.50 (0.70, 8.94)	
4^th ^	9	29.03	46	20.54	3.23 (0.94, 11.12)		2.97 (0.84, 10.56)	
Total	31	100	224	100				
Variables	Case		Control		Crude OR^a^	p for trend	Adjusted OR	p for trend ^b^
	n	%	n	%	(95% CI)		(95% CI)b	
Total colorectal cancers								
*H. pylori* infection status ^c^								
Negative (<0.8)	28	30.77	90	40.18	1.00 (reference)	0.053	1.00 (reference)	0.115
Equivocal (0.8-1.2)	13	14.29	39	17.41	1.07 (0.50, 2.29)		0.98 (0.45, 2.15)	
Positive (>1.2)	50	54.95	95	42.41	1.69 (0.98, 2.92)$		1.56 (0.88, 2.74)#	
Total	91	100	224	100				
Anti-*H. pylori *IgG concentration quartiles ^d^								
1^st ^	15	16.48	66	29.46	1.00 (reference)	0.013	1.00 (reference)	0.049
2^nd ^	25	27.47	60	26.79	1.83 (0.88, 3.80)		1.70 (0.81, 3.60)	
3^rd ^	24	26.37	52	23.21	2.03 (0.97, 4.26)		2.01 (0.94, 4.31)	
4^th ^	27	29.67	46	20.54	2.58 (1.24, 5.39)		2.14 (1.00, 4.58)	
Total	91	100	224	100				

^a^ OR, odds ratio; 95% CI, 95% confidence interval; ^b^ Adjusted for age groups (0-29, 30-39, 40-49, 50-59, 60-69, ≥70 ages), sex, BMI (18.5 to <23, 23 to <25, ≥25, <18.5 kg/m2), an education level (Primary school or under, secondary school, high school, higher high school, unknown), and lifetime smoking (yes/no), total fruits intake (Tertile), total vegetables intake (Tertile), total meats intake (Tertile), total fishes intake (Tertile); ^c ^Classification according to the manufacturer's instructions; ^d^ Classification according to anti-H. pylori IgG concentration of the Cut-off Index (COI) qualitative

## Results

The proportion of men was 64.44% of CRC cases and 65.18% of hospital controls. Both cancer cases and controls aged from 20-29 to 70 or older. Participants had mainly completed junior high school (51.65% cancer cases and 47.32% control cases). The proportion of obese (BMI =/>25) was 8.79% in cancer cases and 10.27% of control cases (Data not presented). 

The mean (± standard deviation, SD) of the COI qualitative for the groups of negative (COI<0.8), equivocal (COI of 0.8-1.2), and positive (COI>1.2) were 0.50 ± 0.16, 1.00 ± 0.11, 2.35 ± 0.93, respectively. That was for the lowest quartile (the mean+/-SD) 0.42 ± 0.11) and the highest quartile as high as 3.08 ± 0.76), Table 1.

Overall 54.95% of the CRC cases and 42.41% of the controls were *H. pylori*-seropositive, Compared to individuals who negative with *H. pylori*, *H. pylori-*positive patients showed the greater odds of CRC (OR (95%CI): 1.56 (0.88, 2.74), p for trend=0.115). Positive dose-response association in quartiles, highest vs lowest, was observed for total CRC, (OR (95%CI): 2.14 (1.00, 4.58), p for trend=0.049), Table 2. 

For the association between H. pylori infection and the subgroups of CRC, positive versus negative, the ORs were 1.52 (95%CI: 0.77, 3.01, p for trend=0.212) for colon cancer; 1.74 (95%CI: 0.53, 5.70, p for trend=0.409) for proximal colon; 1.54 (95%CI: 0.84, 2.81, p for trend=0.134) for distal colon and rectum cancers combined; 1.60 (95%CI: 0.68, 3.76, p for trend=0.267 for rectal cancer, Table 2. By sex, the positive versus negative, the odds of CRC was 1.57 (95%CI: 0.75, 3.28, p for trend=0.129) for men; and 1.51 (95%CI: 0.59, 3.89, p for trend=0.312) for women (Data not presented). None of these odds ratio and 95% confidence interval was statistically significant. 

Regarding the dose-response association in quartiles of *H. pylori* and CRC, highest compared to lowest, the odds of cancer were 1.52 (95%CI: 0.37, 6.25, p for trend=0.571) for proximal colon; 2.38 (95%CI: 1.03, 5.50), p for trend=0.039) for distal colon and rectum cancers combined; 2.97 (95%CI: 0.84, 10.56), p for trend=0.129) for rectum cancer. By sex, highest vs lowest, the odds of CRC was 1.80 (95%CI: 0.65, 4.93, p for trend=0.234) for men, and 2.49 (95%CI: 0.73, 8.50, p for trend=0.109) for women (Data not presented). 

## Discussion

In this case-control study, we observed a non-significant positive association between *H. pylori* seropositive and CRC (positive versus negative) but the significant positive dose-response association by COI qualitative in quartiles (the highest vs the lowest quartile). The significant positive dose-response association in quartiles between *H. pylori* infection and distal colon and rectum cancers combined was stronger than proximal colon cancer. The findings support the hypothesis that *H. pylori* infection might play a role in the development of CRC. 

*H. pylori* is a 2.5-3 μm long twisted or helical gram-negative germ responsible for the development of gastric cancer and duodenal ulcers. Chronic infection with *H. pylori* is carcinogenic to humans (Group 1), according to the evaluation by the International Agency Research on Cancer. *H. pylori* infection induces non-cardiac gastric cancer and mainly targets in the lower one-third of the stomach. *H. pylori* appear to have a different relationship with gastric carcinoma arising in the region of the distal stomach to cardia (non-cardia gastric carcinoma) compared with the cardia region located from adjacent to esophageal sphincter (IARC, 2012). It indicated that the risk of stomach cancer due to *H. pylori* infection has differed from the anatomical location. Therefore, in the same way, *H. pylori* infection might affect colorectal cancers by a specific anatomical location. The previous findings have supported our findings, that is, there was a significant positive association between *H. pylori* infection and distal colon but not or small association with the proximal or the rectum cancer (Buso et al., 2009; Inoue et al., 2011; Zhang et al., 2012). Because gram-negative bacteria of *Fusobacterium, Escherichia/Shigella*, and *Leptotrichia *are common colonization in the left colon (Kim et al., 2018), including the distal colon, therefore, as the same group of gram-negative, *H. pylori* might colonize in this part of colorectal. For the *Shigella* family, Shigellosis is an infection of the intestines caused by *Shigella* bacteria. The recommended course of treatment for the Shigellosis infection of the intestines is for 5 days (Ministry of Health, 2016), while the standard triple therapy to eradicate *H. pylori* infection, including a proton pump inhibitor combined with clarithromycin and amoxicillin or metronidazole, has been recommended for two weeks (Nguyen et al., 2008). Due to common antibiotic overuse, a structure of the distal microbiome might be damaged while *H. pylori* infection will chronically colonize and possibly induce cancer in this distal colon. The previous findings supported this explanation, that is, antibiotic use increased the risk of colon cancer but not rectal cancer (Kilkkinen et al., 2008; Zhang et al., 2019). However, these two studies have not yet examined for the sub-site of proximal and distal colon cancer. 

To date, the underlying causation of the association between *H. pylori* infection and CRC remains unclear. Investigators have hypothesized that potential oncogenic interactions between *H. pylori* and CRC mucosa, including induction of inflammatory responses, modification of gut microflora, and release of toxins which may induce cancer formation (Papastergiou et al., 2016; Terzic et al., 2010). 

Our findings are consistent with the results of the positive association between serologic responses to *H. pylori* proteins and CRC (Butt et al., 2019; Epplein et al., 2013). The results are also consistent with the recent Meta-analyses confirmed the positive association between *H. pylori* infection and the development of CRC (Chen et al., 2013; Guo & Li, 2014; Hong et al., 2012; Rokkas et al., 2013; Wu et al., 2013; Yang et al., 2019; Zhao et al., 2016; Zhao et al., 2008; Zumkeller et al., 2006), and the findings of the original study in Germany (Zhang et al., 2012), in Korea (Park et al., 2018), and China (Liu et al., 2019). The available Meta-analysis by 2019 has not examined for the subgroups of distal and rectum cancers combined. The findings from a large population-based case-control study in Germany have supported the present study results (Zhang et al., 2012), that is, there was a null but significant positive association between *H. pylori* infection and colon cancer and distal and rectum cancers combined, respectively. 

Our study has some strengths. We collected information on smoking and diet to control for confounding in data analysis. Both serum of cancer cases and controls was examined by the same methods and at the same time and laboratory conditions for reliable comparison of the status of *H. pylori* infection indicators between the cancer cases and the controls. We were also able to examine the anatomical subgroups to explore the differences in *H. pylori* infection across the lower gastrointestinal tract.

Our study has certain limitations. The sample size is limited, therefore, the power of the study is being decreased in the subgroups of the study population of colon and rectum cancers. The indicators of serologic responses to *H. pylori* proteins are not available that were associated with the increased risk of CRC in the previous studies (Butt et al., 2019; Epplein et al., 2013). *H. pylori* IgG antibody in serum might not represent the colonization of *H. pylori* in the CRC tumor.

In summary, there was a positive dose-response in quartiles for total CRC and the subgroup of the distal colon and rectum cancers combined. Further studies are needed to determine the mechanistic association between CRC and *H. pylori* infection. 
